# Anti-PD-1 immune-related adverse events are associated with high therapeutic antibody fixation on T cells

**DOI:** 10.3389/fimmu.2022.1082084

**Published:** 2022-12-20

**Authors:** Marianne Gazzano, Christophe Parizot, Dimitri Psimaras, Aurore Vozy, Marine Baron, Baptiste Abbar, Vincent Fallet, Elena Litvinova, Anthony Canellas, Cristina Birzu, Valérie Pourcher, Mehdi Touat, Nicolas Weiss, Sophie Demeret, Damien Roos-Weil, Jean-Philippe Spano, Celeste Lebbe, Joe-Elie Salem, Jacques Cadranel, Baptiste Hervier, Guy Gorochov, Amélie Guihot

**Affiliations:** ^1^ Department of Immunology, Pitié Salpêtrière Hospital, Hospital, Assistance Publique-Hôpitaux de Paris (AP-HP) Sorbonne Université, Paris, France; ^2^ Department of Neuro-Oncology, Pitié Salpêtrière Hospital, Assistance Publique-Hôpitaux de Paris (AP-HP) Sorbonne Université, Paris, France; ^3^ Department Medical Oncology, Pitié Salpêtrière Hospital, Assistance Publique-Hôpitaux de Paris (AP-HP) Sorbonne Université, Paris, France; ^4^ Department of Pneumology and Thoracic Oncology, Tenon Hospital, Assistance Publique-Hôpitaux de Paris (AP-HP) Sorbonne Université, Paris, France; ^5^ Department of Neurology 2-Mazarin, Pitié Salpêtrière Hospital, Assistance Publique-Hôpitaux de Paris (AP-HP) Sorbonne Université, Paris, France; ^6^ Pitié Salpêtrière Hospital, Assistance Publique-Hôpitaux de Paris (AP-HP) Sorbonne Université, INSERM, CNRS, UMR S 1127, Institut du Cerveau et de la Moelle épinière, Paris, France; ^7^ Service des maladies infectieuses et tropicales, Pitié Salpêtrière Hospital, Assistance Publique-Hôpitaux de Paris (AP-HP) Sorbonne Université, Paris, France; ^8^ INSERM UMR-S 1136, Pierre Louis Institute of Epidemiology and Public Health, Sorbonne Université, Paris, France; ^9^ Department of Neurology, Médecine intensive – réanimation à orientation neurologique, Pitié-Salpêtrière Hospital, Assistance Publique-Hôpitaux de Paris (AP-HP) Sorbonne Université, Paris, France; ^10^ Clinical Hematology Unit, Pitié-Salpêtrière Hospital, Assistance Publique-Hôpitaux de Paris (AP-HP) Sorbonne Université, Paris, France; ^11^ Department of Dermatology, Saint Louis Hospital, APHP, Paris, France; ^12^ Department of Pharmacology, Cardio-oncology Program, CIC-1901, Pitié Salpêtrière Hospital, Assistance Publique-Hôpitaux de Paris (AP-HP) Sorbonne Université, Paris, France; ^13^ Department of Internal Medicine, Saint Louis Hospital, Assistance Publique-Hôpitaux de Paris (AP-HP), Paris, France; ^14^ Centre d'Immunologie et des Maladies Infectieuses (CIMI-Paris), INSERM U1135, CNRS ERL8285, Sorbonne Université, Pitié-Salpêtrière Hospital, Paris, France

**Keywords:** biomarker, checkpoint inhibition, irAEs, immune related adverse events, flow cytometry, prediction

## Abstract

Immune checkpoint inhibitors (ICI) widely improved the treatment of solid and hematologic malignancies. Yet, a remarkable proportion of patients receiving ICI develop immune related adverse events (irAEs) which are difficult to define as treatment-related. This underlines the need to develop a biomarker to guide irAE diagnosis. We developed a novel flow cytometry assay combining measurement of anti-PD-1 (programmed cell death protein-1) occupancy and evaluation of remaining PD-1 receptor availability with anti-IgG4 PE and anti-PD-1 BV421. We prospectively collected blood and biological fluids samples from patients treated by IgG4 anti-PD-1 therapy (nivolumab or pembrolizumab), with (n=18) or without (n=12) current irAE. We analyzed PD-1+ and IgG4+ staining pattern and MFI values of these parameters on CD4 and CD8 T cells, and IgG4+/PD-1+ MFI ratios are calculated. A higher mean fluorescence intensity IgG4+/PD-1+ ratio was measured on peripheral CD4+ T cells of irAE cases, when compared to controls (p=0.003). ICI-related toxicity is therefore associated with increased therapeutic antibody occupancy of PD-1 receptors on CD4+ T cells. Furthermore, in one case of ICI-related pneumonitis, binding of therapeutic antibody was stronger on lung CD4+ T cell than in blood. In another case of ICI-related encephalitis, the PD-1 receptor occupancy was total on CSF CD4 T cells, but only partial on peripherical CD4 T cells. Our results suggest that flow cytometry monitoring of ICI occupancy can be used in patients treated with monoclonal ICI to guide irAE diagnosis.

## 1 Introduction

Immune checkpoint inhibitors (ICI) targeting programmed cell death protein (PD-1) is currently used for the treatment of a wide range of solid and hematologic malignancies. Four fully human monoclonal anti-PD-1 antibodies of the Immunoglobulins G 4 subclass (nivolumab, pembrolizumab, cemiplimab and spartalizumab) have been approved by the Food and Drug Administration and European Medicine Agency for the treatment of, amongst others, advanced stages of non-small-cell lung cancer, melanoma, head and neck squamous cell carcinoma and renal-cell cancer, in both first and second-line therapy. They produce durable objective responses and demonstrate benefits in the survival rate of treated patients (1). Because ICI have no direct effect on the tumor itself, but rather allow a spectacular re-activation of exhausted T cell lymphocytes, 14% of treated patients develop T cell-mediated high grade immune related adverse events (irAEs) that can affect almost any tissues such as gut, skin, endocrine glands and lungs, and can occur even years after treatment (2). Although anti–PD-1 agents are usually better tolerated than ICIs targeting CTLA-4, they can be fatal to ~1% of patients receiving the former antibodies (2). Discontinuation of treatment should therefore be considered in the event of anti–PD-1-mediated irAEs, although immune related adverse effects have been associated with improved outcomes in cancer patients treated by anti-PD-1 (3).

The pathophysiology of irAEs induced by immune checkpoint blockade remains unclear. Potential mechanisms have been described, showing enhanced Th1 and Th17 cell responses during the development of irAEs with the production of pro-inflammatory cytokines (IL-6, IL-17, TNFα and IL-1β) in colitis induced by ICI (4, 5). ICI can also alter T cell–B cell interactions resulting in pathogenic autoantibody production in thyroid disorders (6). The appearance of the inflammatory infiltrate with predominance of CD8+ cells in ICI-induced myositis and myocarditis has been shown (7, 8).

Up until now it remains difficult to define an undesirable event as treatment-related, as it is mainly an exclusion diagnosis that requires elimination of sepsis (bacterial or fungal) or tumour progression. It is indeed unclear whether plasma concentrations of nivolumab correlate with irAEs (1). While cellular biomarkers associated with treatment efficacy have been reported (9), a reliable biomarker for anti–PD-1-mediated irAE remains needed considering the increasing numbers of ICI-treated patients.

Osa et al. detected sustained nivolumab binding on T cells that may be responsible for residual efficacy in regard to both activated antitumor immunity and autoimmunity (10). They confirmed that PD-1-occupancy remained detectable more than 20 weeks after the last infusion by therapeutic antibody on T cells and that Ki-67+ T-cells significantly correlated with clinical response to nivolumab treatment. We tested a modified version of this flow cytometry approach in order to determine whether PD-1 occupancy levels and/or Ki67 expression on T cells might also be correlated with irAE.

## 2 Materials and methods

### 2.1 Patients

Fresh samples of patients treated by IgG4 subtype immune checkpoint inhibitors (nivolumab, pembrolizumab or spartalizumab) were routinely collected in the APH-HP Sorbonne Université (Pitié Salpêtrière, Tenon) and Saint Louis Hospital between May 2018 and March 2022 (certification ISO 15189).

Blood samples collected from healthy donors were also analyzed to determine staining pattern standards and competitive binding assays.

Blood (n=31), bronchoalveolar lavage (n=2) and cerebro-spinal fluid (n=2) samples were prospectively collected in patients with or without confirmed irAE and stratified in “toxicity” or “non-toxicity” groups, respectively, regardless of the duration or type of treatment and at the time of irAE (grades 3 to 5 according to Common Terminology Criteria for Adverse Events grading (CTCAE) V5.0. 2017) (11).

Toxicity and non-toxicity groups were compared. Sensitivity describes the fraction of “toxicity” samples accurately identified by tested biomarkers, while specificity describes the fraction of “non-toxicity” samples identified as such.

### 2.2 Flow cytometry

Step-by-step protocols for preparing and analyzing clinical samples by flow cytometry T, B and NK cell phenotypic analysis and numeration was performed on blood samples using an AQUIOS flow cytometer (Beckman Coulter^®^). One mL of freshly collected blood was washed two times with 10 mL phosphate-buffered saline solution (PBS), or with NaCl 0.9% for bronchoalveolar lavage samples. One hundred μL of NaCl 0.5% bovine serum albumin washed blood or washed bronchoalveolar cells were probed 15 minutes in darkness at room temperature (RT) with anti-human CD45-KO (Beckman Coulter^®^), CD3 APC H7 (BD Biosciences^®^), CD4 APC (BD Biosciences^®^), CD8 Alexa Fluor 700 (Beckman Coulter^®^), HLA-DR ECD (Beckman Coulter^®^), CD38 PC5.5 (Beckman Coulter^®^), CD14 PC7 (Beckman Coulter^®^), IgG4 PE (clone HP6025, Southern Biotech^®^) and PD-1 BV421 (Mouse Anti-Human CD279, clone EH12.1, BD Biosciences^®^). Samples were then washed with 2 mL PBS and incubated for 45 minutes RT with 300μL fixation/permeabilization buffer (Thermo Fisher Scientific, eBioscience^®^ Foxp3/Transcription Factor Staining Buffer Set) for intracellular staining. Samples were washed with 1 mL permeabilization buffer and after removing supernatant, 100 μL permeabilization buffer was added. Samples were then probed for 15 minutes RT with anti-Ki67 FITC (BD Biosciences^®^) followed by 1mL PBS washing. Cells were analyzed in 300 μL PBS using a Navios cytometer (Beckman Coulter^®^). Each run was preceded by a standardization (Flow-Set Pro Fluorospheres Beckman Coulter^®^), permitting mean fluorescence intensity interpretation between samples.

### 2.3 Competitive binding assay

To check for potential competition between nivolumab and anti-PD-1 BV421, 100 μL of whole healthy blood donors was saturated with 10 μL (0.1 mg/mL final) of commercial nivolumab (Opdivo^®^, from remaining 1 mg/mL therapeutic medication) for 30 minutes RT. After washing, increasing concentrations of anti-PD-1 BV421 (αPD-1) flow cytometry antibody were added and incubated 30 minutes at RT. After washing, remaining bound nivolumab was then determined by anti-IgG4 staining (clone HP6025) as above.

### 2.4 Statistics

The Mann-Whitney and Chi-square tests were used to assess statistical differences between “toxicity” and “non-toxicity” groups for each biomarker, p-values < 0.05 were considered significant. Discriminatory capability of the features was additionally assessed using ROC-curves and the corresponding area under the curves (AUCs). We report features with AUC > 0.65 as discriminatory. Data analysis was performed with FlowJo V10 (TreeStar) software, GraphPad Prism 8.0.1 software and RStudio 1.3.1093 (Foundation for Statistical Computing). Principal-component analysis (PCA) was performed using RStudio v1.3.1093 with FactoMineR PCA and Factoextra fviz_pca_biplot functions on MFI ratio values.

## 3 Results

### 3.1 PD-1 flow cytometry detection spares therapeutic antibody binding

**Figure 1:**
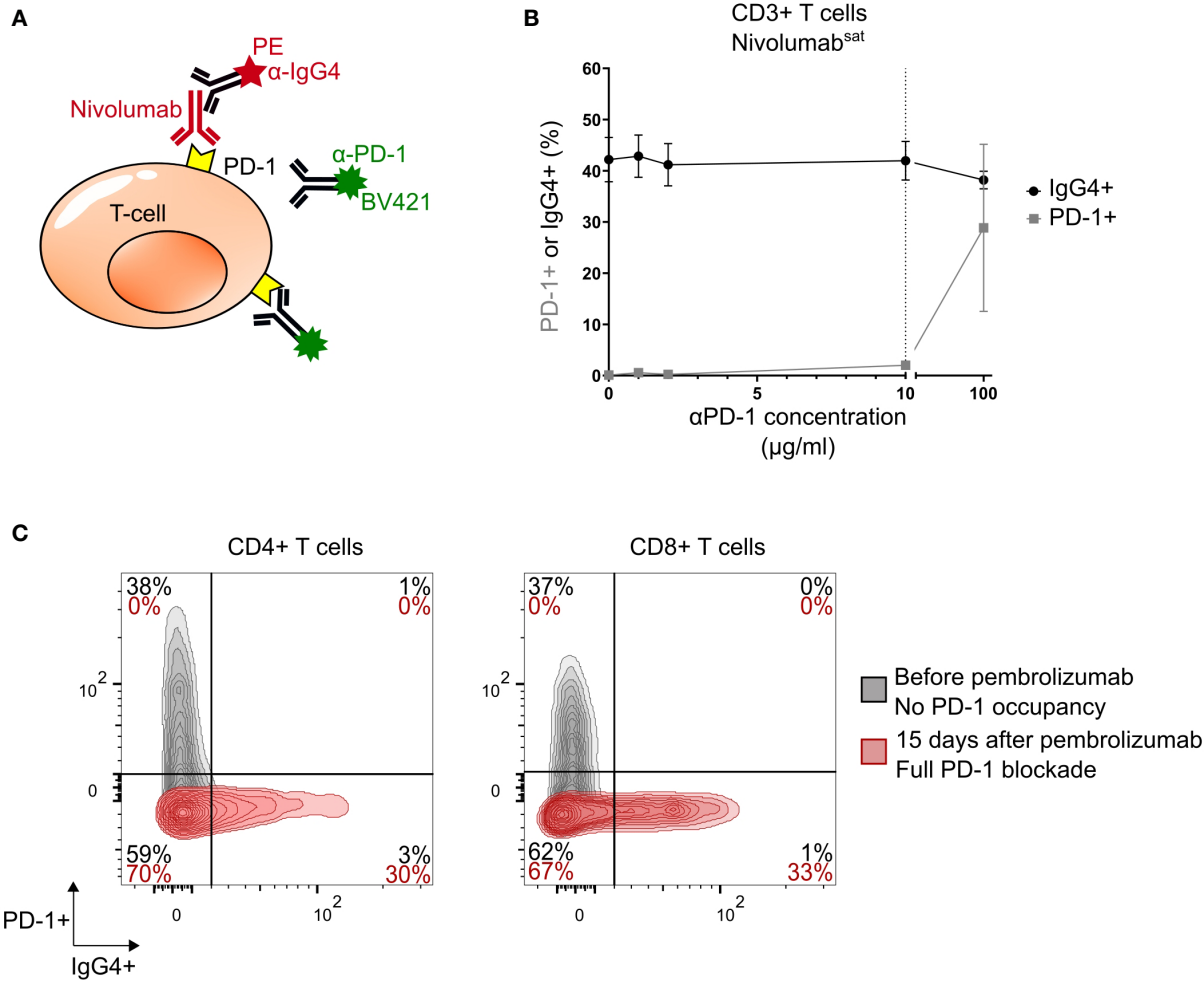
Competitive binding assay and phenotyping example. **(A)** Principle of the experiment. **(B)** Percentage of IgG4+ (black) and PD-1+ (grey) CD3+ T cells (mean ± SEM) after saturation (Nivolumab^sat^= αPD-1^sat^= 1 mg/mL; 10μL for 100μL blood, 100μg/mL final) and adding increasing concentration of αPD-1 antibody on healthy donors’ blood samples (n = 4). **(C)** Flow cytometry analysis of cells from blood of a patient (Pt#1) with PML (Progressive multifocal leukoencephalopathy) before (grey) and 15 days after pembrolizumab treatment initiation (red): IgG4, PD-1, and double staining PD-1+IgG4+ levels in CD4+ (left) and CD8+ (right) T cells. Population percentages. PD-1, Programmed cell death protein 1 receptor; SEM, standard error of the mean.

We developed a flow cytometry monitoring of PD-1 receptor occupancy on freshly collected whole blood, bronchoalveolar lavage or cerebrospinal fluid samples from patients who received anti-PD-1 (nivolumab, pembrolizumab or spartalizumab) as part of their treatment. These molecules are fully human or humanized IgG4 antibodies. We postulated that a novel assay that would combine detection of PD-1-bound therapeutic antibody and remaining unblocked PD-1 receptor levels might best describe status of T cell blockade (**Figure 1A**, **Supplemental Figure 1A**).

First, to determine whether T cells opsonized with therapeutic anti-PD-1 can be analysed with anti-PD-1 flow cytometry antibodies (αPD-1) without displacing the former antibodies, we saturated blood cells of four healthy donors with nivolumab. We then added increasing concentrations of αPD-1 antibody and used an anti-IgG4 antibody to detect remaining bound nivolumab. As shown, addition of αPD-1 antibodies only slightly reduced nivolumab cell-surface positivity from 42.2% (mean, SEM=4.3%) to 42.0% IgG with 10 μg/mL αPD-1, SD=3.8%) among CD3+ cells (mean variation: 0.5%, p>0.99) (**Figure 1B**, **Supplemental Figures 1B, C**). In contrary, higher concentration of PD-1 displaces the therapeutic antibody. These results confirm that 10 μg/mL αPD-1 antibody does not significantly displace cell-bound nivolumab, and therefore can be used as a biomarker of *in vivo* remaining PD-1 availability upon nivolumab exposure.

We longitudinally monitored PD-1 expression on T cells from a patient with progressive multifocal leukoencephalopathy before and after pembrolizumab introduction. As shown, surface PD-1 is detected by αPD-1-BV421 staining before ICI treatment (39% and 37% of PD-1+ cells among CD4+CD3+ and CD8+CD3+ cells, respectively), but not anymore 15 days after pembrolizumab introduction (**Figure 1C**). Conversely, cell-bound pembrolizumab is revealed by anti-IgG4 staining at the late time point of analysis (31% and 33% of pembrolizumab+ cells, **Figure 1C**). Thus, 15 days after treatment onset, PD-1 surface receptors of circulating CD4+ and CD8+ T cells appear totally saturated or modulated by pembrolizumab. This cytometry analysis allows to define three PD-1 occupancy profiles: no binding, partial binding and complete binding (**Supplemental Figures 1B, C**).

### 3.2 High ratio of PD-1 blockade is associated with ICI-related toxicity

**Figure 2:**
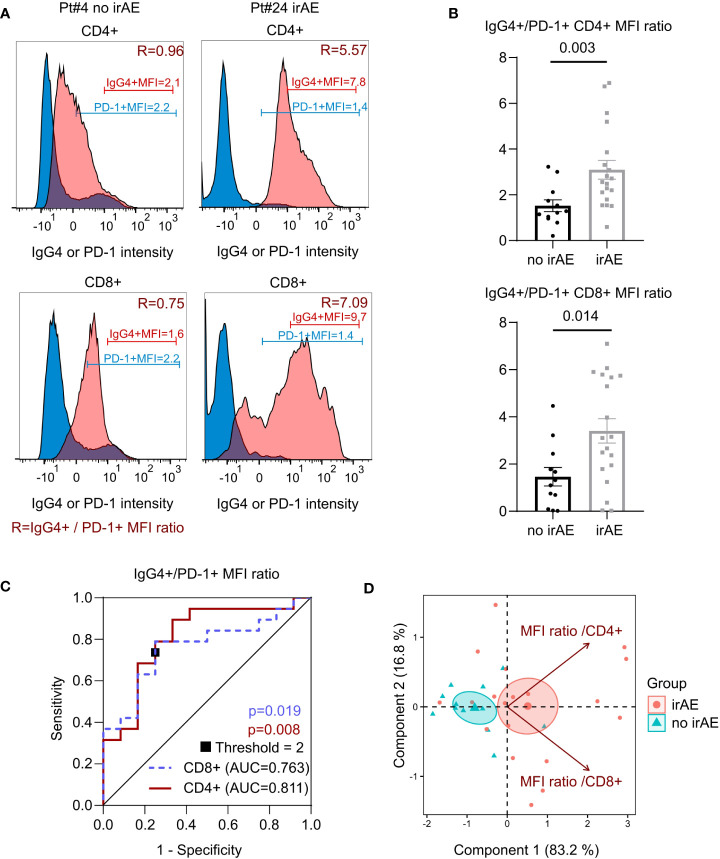
Comparison of therapeutic antibody binding during ICI (Immune checkpoint inhibitors) related toxicity (irAE). **(A)** Representative example of flow cytometry analysis of cells from blood of two patients with (Pt#24, myocarditis) or without side effect (Pt#4) post ICI (both). Histograms of IgG4 and PD-1 levels in CD4+ and CD8+ T cells. IgG4+ MFI, PD-1+ MFI and MFI ratio (shown in dark red, R). **(B)** Ratio of mean fluorescence intensity (mean ± SEM) of IgG4+ and PD-1+ in CD4+CD3+ cells and CD8+CD3+ cells of patients treated by ICI developing (n=18) or not irAEs (n=12). Statistical test: Mann-Whitney test. **(C)** Receiver operating characteristic curves of MFI ratio: CD8 (blue dashed line), CD4 (red line), MFI ratio threshold (square). Statistical test: Chi-square. **(D)** Principal component analysis of IgG4+ to PD-1+ MFI ratios in CD4+ or CD8+ T cells in patients with or without irAE (n= 30). The biplot indicate frequencies of samples described by each principal component. MFI ratios are represented by vectors. Ellipses represent the 95% CI of patient distribution in each group. Center of ellipse is indicated.

Patients without (n=12) or with confirmed irAE (n=19) of various toxicity types affecting different organs were compared (**Supplemental Table 1**). In the irAE cases included in this analysis, lack of tumour progression was verified by biopsy sampling for immunofluorescence analysis, as recommended by published guidelines (12), and infectious complications were excluded. Since percentages of nivolumab-bound T cells varied between patients (19.2% and 19.7% SD for CD4+ and CD8+ T cells, respectively), we based our comparisons on Mean Fluorescence Intensity values. As shown, the IgG4/PD-1 MFI ratio of CD4+ T cells is significantly increased in irAE when compared to non irAE cases (mean 3.10 vs. 1.52, P = 0.003, **Figure 2B**). The CD8+ IgG4/PD-1 MFI ratio was also increased in irAE patients (mean 3.41 vs 1.46, P = 0.014, **Table 1** and **Figure 2**). Importantly, median time from last infusion was higher in the irAE group compared to the other one (37 vs. 16 days; P = 0.016, **Supplemental Table 1**), therefore higher IgG4/PD-1 MFI ratios in irAE cases cannot be related to more recent therapeutic infusion in this group. Selecting a MFI ratio threshold value of 2 discriminates the two groups significantly in CD4+ (P=0.008; OR=8.4; AUC=0.811) and in CD8+ T cells (P=0.019; OR=6.5; AUC=763) with sensitivities of 82% and 81%, respectively, and specificities of 64% and 60%, respectively (**Figure 2C**). The positive predictive value of MFI ratio value superior to 2 is 74% in CD4+ and 68% in CD8+.

**Table 1:** Percentage of proliferation and activation markers positivity in total and therapeutic antibody-bound CD4 and CD8 T cells from patients with or without immune related adverse events.

	Parameter	Median (SD) no irAE (n=12)	Median (SD) irAE (n=19)	P-value
**CD4+ T cell**	Ki67+ (%)	3.89 (3.31)	3,47 (4.10)	0.881
	HLA-DR+ (%)	21.61 (17.14)	24.44 (17.38)	0.734
	PD-1+ (%)	6.60 (9.40)	2.89 (12.15)	0.921
	IgG4+ (%)	27.50 (18.10)	32,75 (20.37)	0.952
	IgG4/PD-1 ratio	7.56 (33.28)	4.80 (78.07)	0.921
	PD-1+IgG4+ (%)	4.83 (5.60)	1.79 (7.45)	0.667
	IgG4+/PD-1+MFI ratio	1.25 (1.06)	2.56 (1.84)	0.003
	IgG4+Ki67+ (%)	2.75 (177.54)	2.02 (4.04)	0.459
	PD-1+Ki67+ (%)	0.53 (1.48)	0.46 (3.12)	0.772
				
**CD8+ T cell**	Ki67+ (%)	2.57 (4.76)	2.61 (7.63)	0.646
	HLA-DR+ (%)	65.8 (18.48)	66.67 (21.23)	0.734
	CD38+ (%)	66.84 (17.42)	71.77 (20.34)	0.265
	PD-1+ (%)	5.27 (16.16)	4.09 (17.08)	0.638
	IgG4+ (%)	38.56 (16.81)	45.73 (21.13)	0.252
	IgG4/PD-1 ratio	4.97 (64.89)	5.49 (42.27)	0.818
	PD-1+IgG4+ (%)	4.89 (10.67)	3.33 (12.26)	0.984
	IgG4+/PD-1+ MFI ratio	1.21 (1.36)	3.10 (2.25)	0.014
	IgG4+Ki67+ (%)	2.06 (6.18)	1.47 (4.60)	0.984
	PD-1+Ki67+ (%)	0.13 (3.26)	0.13 (5.75)	0.755

Mann Whitney test.

In return, we found no significant difference in T cells (CD3+CD4+ or CD3+CD8+), B cells (CD3-CD19+) or NK cells (CD3-CD16+CD56+) absolute counts between the two groups (**Supplemental Table 1**). The proportion of Ki67+, HLA-DR+, CD38+, PD-1+ expression or nivolumab occupancy and PD1+/IgG4+ percentages ratio were neither significantly different between toxicity groups (**Table 1**). Finally, T cells blocked by therapeutic antibody (IgG4+) or therapeutic antibody-free (PD-1+) expressed similar proportions of the Ki67 activation marker. Thus, only IgG4/PD-1 MFI ratio calculation, and not comparison of the various independent markers tested allowed phenotypic discrimination between toxicity groups.

We performed principal component analysis (PCA) to determine the relative contribution of PD-1 blockade on CD4+ or CD8+ T cells to irAEs. As shown (**Figure 2D**) PCA discriminates in irAE negative and irAE positive patients with equal contribution of CD4+ and CD8+ MFI ratios. Interestingly, PD-1+ MFI ratios are typically different in CD4 and CD8 subsets of a given patient, as can be observed in the PCA of **Figure 2D**.

### 3.3 Exacerbated PD-1 blockade in lung and CSF in patients with irAEs

**Figure 3:**
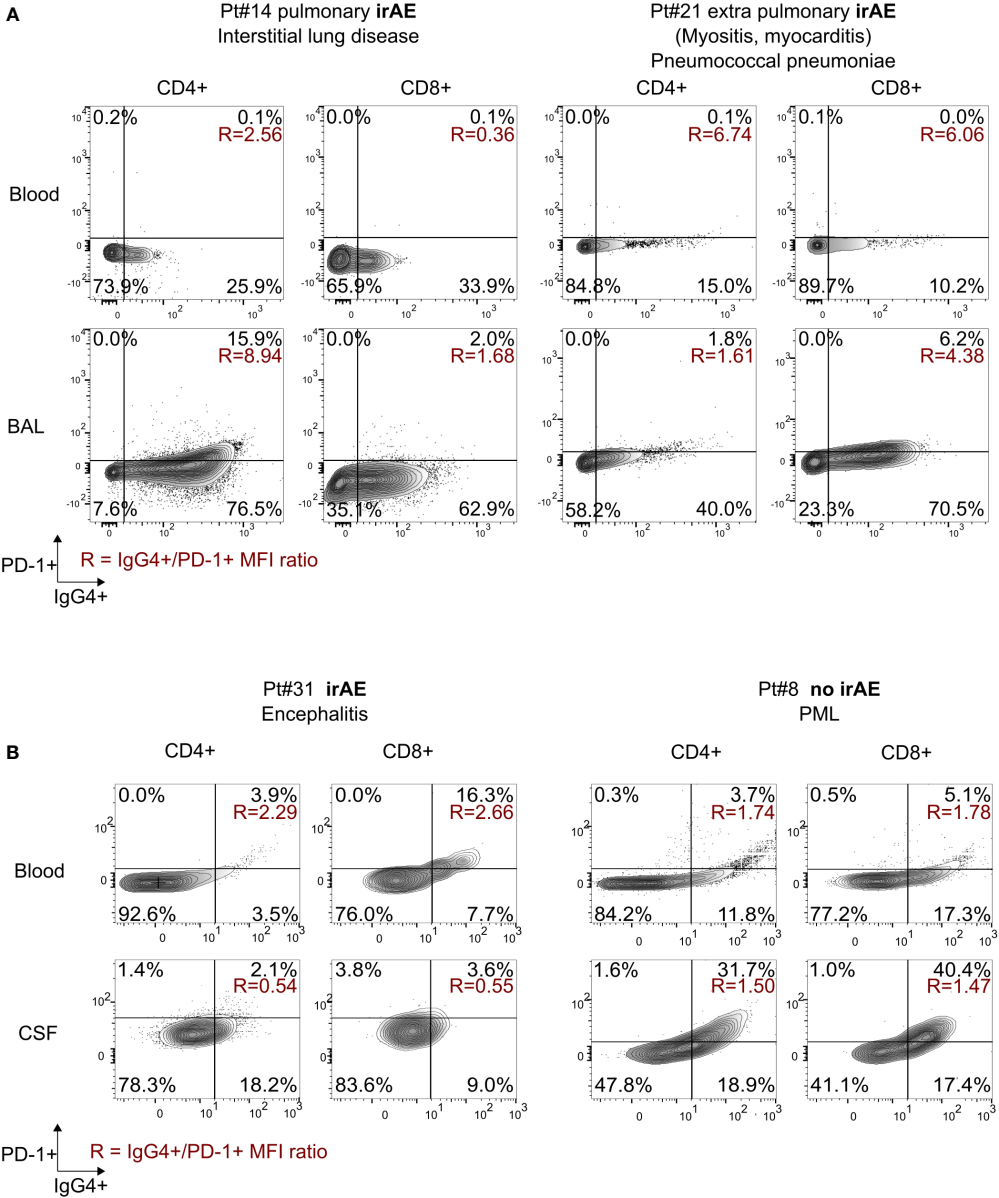
Flow cytometry analysis in other body fluids. **(A)** Analysis of cells from BAL and blood of two patients with pulmonary affection, one related to ICI and one non-ICI related; **(B)** analysis of cells from CSF and blood of two patients treated with pembrolizumab for PML (Pt#8) or colorectal cancer with encephalitis (Pt#31). IgG4, PD-1, and double staining PD-1+IgG4+ levels in CD4+ and CD8+ T cells. Population percentages*;* IgG4+ to PD-1+ MFI ratios are shown in red. BAL, bronchoalveolar lavage; CSF, cerebrospinal fluid.

Bronchoalveolar lavage (BAL) and blood T cells from two patients with ICI-related pulmonary toxicities were also analysed. Pt#14 suffered from ICI-induced pneumonitis. Pt#21 suffered from myositis and myocarditis. Pt#21 therefore presented with an extra-pulmonary irAE and underwent BAL for pneumococcus pneumoniae. Alveolar lymphocytes represented 20% and 2% of total BAL cells in Pt#14 and 21 respectively. Both patients were treated for renal cancer with nivolumab. Pt#14 required oxygen therapy because of SpO2 88% (bilateral ground-glass opacity on CT scan) and Pt#21 required methylprednisolone. Both patients showed high IgG4 to PD-1 MFI ratio on CD4 blood T cells (2.6 and 6.7 respectively). We found very high IgG4+ to PD-1+ MFIs ratio in CD4+ T-cells in BAL from patient with pulmonary immune toxicity compared to patient with infectious pneumoniae (8.9 vs. 1.6). Proportions of BAL IgG4+ cells among CD4+T cells were also increased (92.4% vs. 26.0%) and presented with a “partial binding profile” (IgG4+PD-1+) in BAL when compared to blood, in Pt#14 with pulmonary toxicity related to ICI (**Figure 3A**).

We also compared blood and cerebrosplinal fluid (CSF) samples from a patient with irAE encephalitis treated for colorectal cancer (Pt#31) and a patient with non irAE-related or JC-virus related progressive multifocal leukoencephalopathy (Pt#8), both treated with pembrolizumab. We found high IgG4+ to PD-1+ MFIs ratio in blood T-cells in patient with neurologic toxicity compared to patient without irAE (2.3 vs. 1.7 in CD4+ and 2.7 vs. 1.8 in CD8+). Interestingly we observed a “complete binding” status (IgG4+PD-1- 18% in CD4+ and 9% in CD8+) in CSF from patient with irAE. Unlike in CSF, a “partial binding” profile was observed on peripheral T cells of the patient with irAE (**Figure 3B**). In contrast, CSF and blood from the non-irAE patient showed a “partial binding” profile. This reflects an exacerbated binding of pembrolizumab on CSF T-cells from a patient with neurologic toxicity. We finally analysed Pt#31 before and after successful treatment with methylprednisolone and therapeutic plasmatic exchange (TPE), resulting in a diminution of CSF pleocytosis (from 58 to 3 lymphocytes per µL after 30 days) and in a shift to a “partial binding” profile in CSF (**Supplemental Figure 2A**), showing the clearance of the therapeutic monoclonal antibody.

We next attempted to analyse whether plasmatic exchange might have an effect on the observed IgG4+/PD-1+ MFI ratio. Pt#15 presented pneumonia after receiving pembrolizumab for non-small cell lung cancer. Pt#21 presented with myositis and myocarditis after receiving nivolumab for renal cancer. TPE was attempted in both cases. Blood samples were collected two days before TPE and immediately after (Pt#15), or 3 days after (Pt#21). After TPE, IgG4 staining on circulating T cells modestly decreased on both CD4 from 34% to 22% (Pt#15) and 15% to 12% (Pt#21) and CD8 from 38% to 37% (Pt#15) and 10% to 3% (Pt#21). Nevertheless, we observed a decrease in CD4 IgG4/PD-1 MFI ratio in Pt#15 (from 3.86 to 0.52) and in Pt#21 (6.74 to 4.86, **Supplemental Figures 2B, C**). Pt#21 passed away three days after TPE and autopsy demonstrated a CD8+ T cell and macrophagic infiltrate organized in tertiary structures in diaphragm, deltoid and quadriceps muscles cryofragments, characteristic of reported immune-related myositis (13). Pt#15 had a pulmonary bacterial secondary infection and passed away a month after TPE and five cures of abatacept (14). These data suggest that therapeutic plasmatic exchange is poorly effective at clearing cell-bound therapeutic monoclonal antibodies.

## 4 Discussion

Diagnosis of immune-mediated adverse effects of immune check point inhibitors represents a clinical challenge for patients and clinicians dealing with solid and hematologic cancers. Here we describe that PD-1 saturation on CD4 T cells, defined with MFIs ratio of IgG4+ (therapeutic antibody) to PD-1 (target), is linked with the occurrence of irAE. Technically, we selected the best anti-PD-1 binder (clone EH12) and found that a final concentration of 10 µg/mL is sufficient to saturate target PD-1 levels on T-cells, suggesting that this concentration is suitable to ensure monitoring of ICI treatment. In addition, we showed that the detection antibody probably shares the same epitope with therapeutic anti-PD-1 antibodies, because high concentration of αPD-1 (100 µg/mL) displaces the therapeutic antibody. However, the concentration used for staining (10 µg/mL) does not displace the therapeutic antibody (**Figure 1B**).

CD4 and CD8 IgG4+/PD-1+ MFI ratio were both the most accurate markers of irAE. Nevertheless, we propose a CD4 mediated mechanism for the pulmonary toxicities we studied. This cell-mediated immune mechanism is also suggested by our results on BAL samples showing strong IgG4 expression on CD4+ T cells. This hypothesis is consistent with previous description of checkpoint inhibitors pneumonitis (CIP) being associated with high frequency of BAL central memory CD4 T cells (15). Of note, patients with grade 5 irAEs (#14, 15, 20 and 21) show a trend toward higher MFI ratio in CD4+ compared to others (median = 3.2 *vs* 2.5, p = 0.59) but the sample size was too small to reach statistical significance. In the present study, there is also evidence of significant difference in therapeutic antibody occupancy profiles in CD8+ T cell between toxicity or no toxicity groups. This is consistent with the high frequency of TNF+CD8+ BAL T cells described during CIP (15). In blood, the IgG4/PD-1 MFI increased ratio was not due to short delays since the time of last anti-PD-1 injection because patients had higher times since last ICI injection in the irAE group.

Thus, exploring the difference of ratio the T cell IgG4/PD-1 MFI ratio between groups may help irAE diagnosis with high sensitivity (82%) and acceptable specificity (64%). Furthermore, monitoring of saturation of antigenic sites in peripheral fluids showed differences with blood saturation profiles. More precisely, we found two different profiles described here: *i)* in BAL of a patient with irAE pneumonitis, we found a strong binding of therapeutic antibody and *ii)* in CSF of a patient with encephalitis, we showed a strong infiltration of IgG4+ lymphocytes compared to blood. Further analysis is required to determine if this difference is linked to irAE occurrence. The T cell lymphopenia we observe in periphery during irAE could reflect tissue migration of IgG4+ T cells detected in BAL and CSF. To go further it would be interesting to increase the cohort size and monitor patients with more various irAE types, because pulmonary irAE are mediated by CD4 T cells but myocarditis/myositis are classically mediated by CD8 T cells (12, 14).

In summary, the results presented here provide a significant basis to develop biomarkers of immune checkpoints inhibitors toxicity in patients with mAb therapy. This monitoring technique of PD-1 antigenic sites saturation by IgG4 mAb could be used to follow patients with monoclonal antibodies immunotherapies to better stratify patients at an earlier time point and guide irAE diagnosis both using peripheral blood and more accurately irAE-targeted tissues.

## Data availability statement

The raw data supporting the conclusions of this article will be made available by the authors, without undue reservation.

## Ethics statement

The study of ICI-treated patients at APHP-Sorbonne Université was reviewed and approved by the central ethic committee of APHP: Comité Scientifique et Ethique (CSE) de l’Entrepôt de Données de Santé de l’AP-HP (EDS AP-HP) under reference CSE-20-37 JOCARDITE (Dec. 25 2022). Flow cytometry clinical data were retrospectively analysed. Written informed consent was obtained from all participants for their participation in this study.

## Author contributions

Conceptualization by AG, CP and MG. MG and AG analysed clinical data. Clinical management performed by AG, BH, JC, J-ES, DP, AV, MB, BA, VF, CL, CB, VP, MT, DR-W. Manuscript organization, writing and editing by MG, AG, GG, CP, BH. All authors had full access to all data in the study and take responsibility for the integrity of the data and the accuracy of the data analysis All authors contributed to the article and approved the submitted version.
