# Supplementary material for: Anti-PD-1 immune-related adverse events are associated with high therapeutic antibody fixation on T cells
Source: Front Immunol. 2022 Dec 20;13:1082084. doi: 10.3389/fimmu.2022.1082084 (PMC9808779; doi:10.3389/fimmu.2022.1082084)
Supplement: Supplementary file 1 [file DataSheet_1.docx]

# Supplementary Material

# Supplementary Figures

**
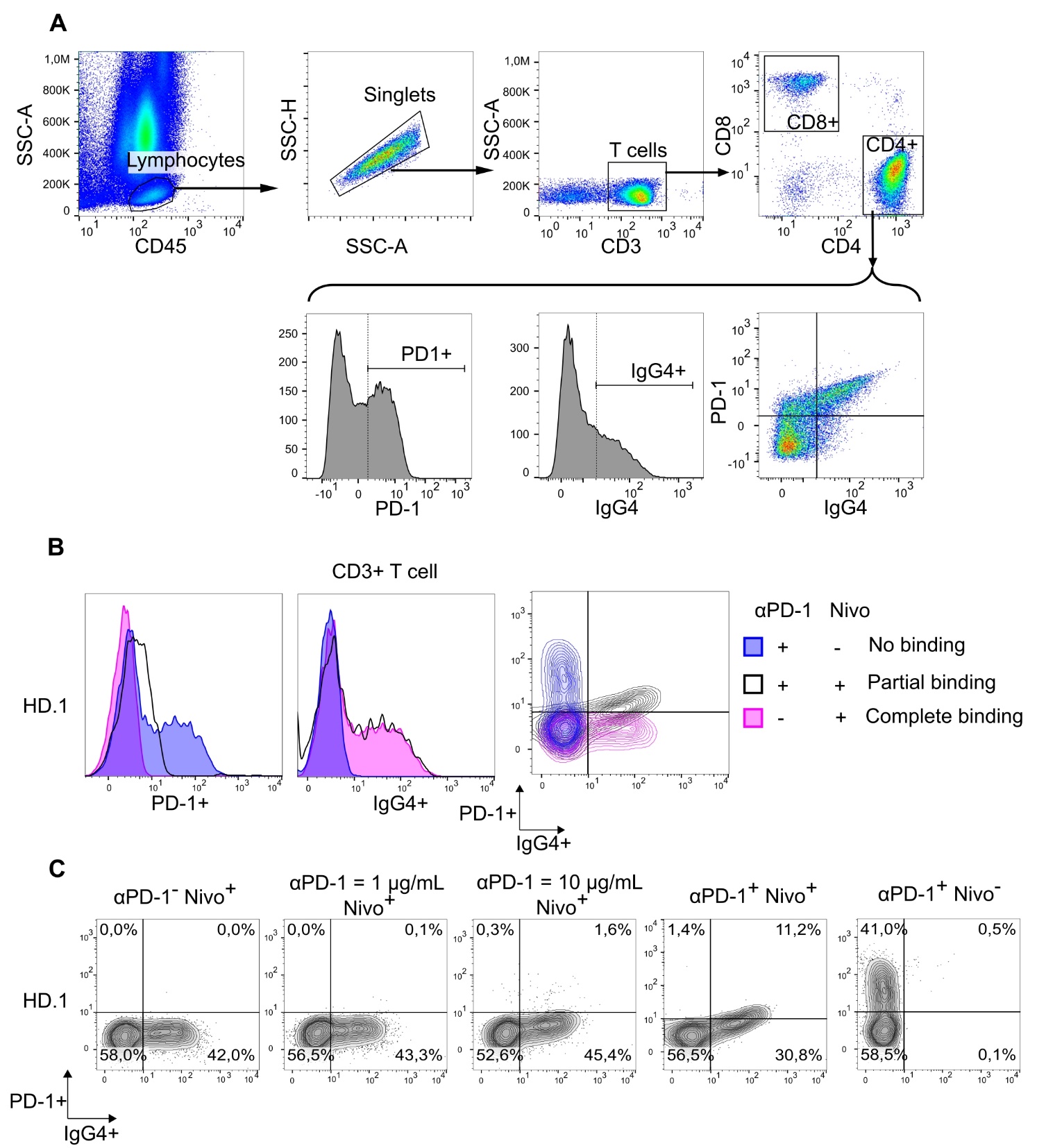
**

# Supplemental figure 1 : Flow cytometry analysis of cells from blood after nivolumab saturation: Histograms and biplot of representative examples in IgG4, PD-1, and double staining PD-1+IgG4+ levels in T cells, in conditions as described in methods. (A) Gating strategy in Pt#24. (B) In healthy donor 1: saturation with αPD-1, no nivolumab (black); αPD-1 excess (100μg/mL) after nivolumab saturation (blue); nivolumab saturation, no αPD-1 (pink)
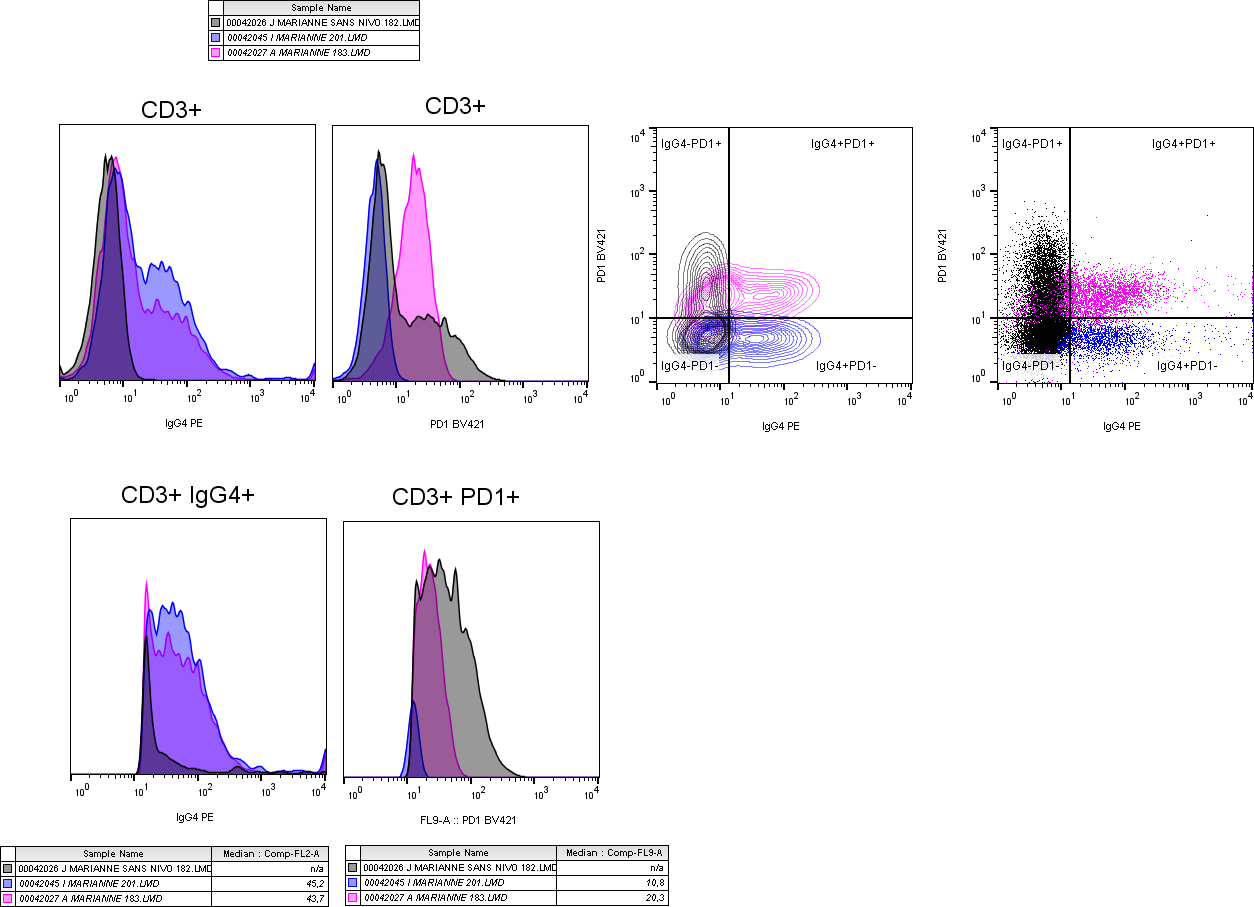
. (C) After nivolumab saturation and adding increasing concentration of αPD-1 in healthy donor 1. Saturated condition αPD-1^+^, nivolumab^+^ =100 μg/mL. PD-1: Programmed cell death protein 1 receptor.


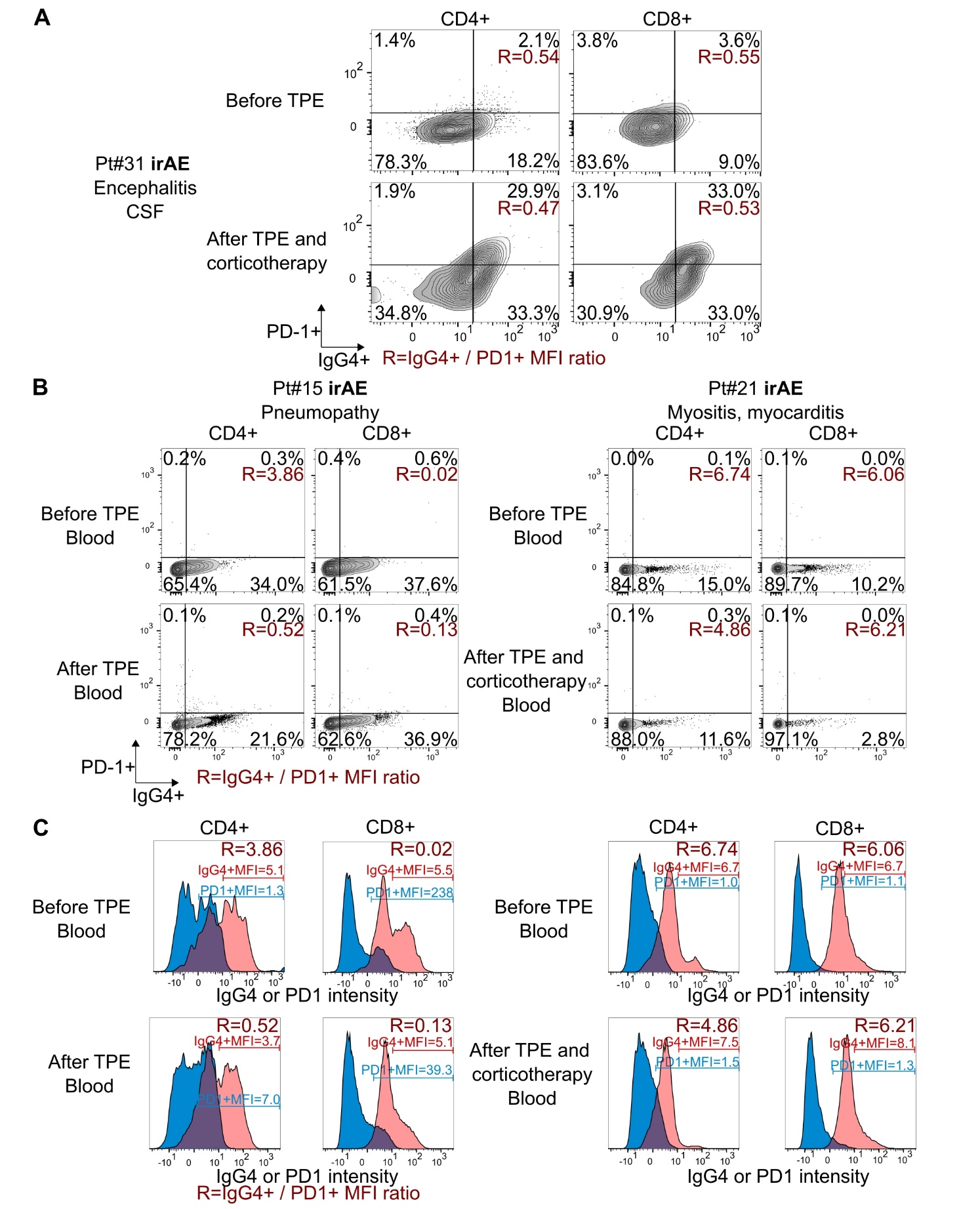


**Supplemental figure 2. Flow cytometry analysis of cells of patients with irAE before and immediately after therapeutic plasmatic exchange.** (**A**) CSF of Pt#31 with encephalitis. (**B**) Blood from Pt#15 with pneumopathy and Pt#21 with myositis. IgG4, PD-1, and double staining PD-1+IgG4+ levels in CD4+ and CD8+ T cells. Population percentages: IgG4+ to PD-1+ MFI ratios are shown in red (R). (**C**) Histograms of IgG4 and PD-1 levels in CD4+ and CD8+ T cells. IgG4+ MFI, PD-1+ MFI and MFI ratio (shown in dark red, R). CSF: cerebrospinal fluid.

# Supplementary Tables

**Supplemental table 1.** Clinical characteristics of patients: tumor type, ICI treatment, toxicity types, and T, B, NK counts at the sample time point.

| **Patient** | **Group** | **Tumor type or Disease** | | | | **Treatment (nb of injections)** | **Toxicity type** | **Time since last injection (days)** | **CD3 (/mm3)** | **CD4 (/mm3)** | **CD8 (/mm3)** | **B (/mm3)** | **NK (/mm3)** |
| --- | --- | --- | --- | --- | --- | --- | --- | --- | --- | --- | --- | --- | --- |
| 1 | no irAE | PML | | | | pembrolizumab (1) | none | 15 | 62 | 14 | 46 | NA | NA |
| 2 | no irAE | Melanoma | | | | nivolumab + ipilimumab (2) | none | 28 | 2493 | 1892 | 591 | 89 | 140 |
| 3 | no irAE | Melanoma | | | | nivolumab + ipilimumab (2) | none | 4 | 1251 | 840 | 378 | 242 | 429 |
| 4 | no irAE | Mesothelioma | | | | nivolumab (2) | none | 17 | 408 | 300 | 110 | 24 | 67 |
| 5 | no irAE | Lung | | | | pembrolizumab (6) | none | 206 | 2257 | 1210 | 1049 | 414 | 171 |
| 6 | no irAE | Lung | | | | nivolumab (1) | none | 20 | 1490 | 709 | 774 | 332 | 208 |
| 7 | no irAE | Lung | | | | nivolumab (NA) | none | 28 | 477 | 411 | 61 | 41 | 88 |
| 8 | no irAE | PML | | | | pembrolizumab (3) | none | 1 | 439 | 259 | 154 | 264 | 103 |
| 9 | no irAE | PML | | | | pembrolizumab (1) | none | 40 | 178 | 129 | 47 | 14 | 114 |
| 10 | no irAE | Lung | | | | pembrolizumab (17) | none | 12 | 1275 | 710 | 542 | 144 | 247 |
| 11 | no irAE | Lung | | | | pembrolizumab (7) | none | 14 | 1042 | 529 | 517 | 544 | 369 |
| 12 | no irAE | Lung | | | | pembrolizumab (28) | none | 7 | 1167 | 755 | 338 | 70 | 104 |
| **Median (SD)** | | | |  |  | 2 (8.6) |  | 16 (56) | 1167 (790) | 709 (529) | 378 (326) | 193 (176) | 156 (118) |
| 13 | irAE | Thymoma | | | | nivolumab (1) | myositis/myocarditis | 24 | 535 | 216 | 302 | 87 | 356 |
| 14 | irAE | Kidney | | | | nivolumab (8) | pneumopathy | 213 | 228 | 95 | 68 | 121 | 14 |
| 15 | irAE | Lung | | | | pembrolizumab (5) | pneumopathy | 37 | 483 | 303 | 161 | 361 | 133 |
| 16 | irAE | Lung | | | | nivolumab (14) | neurologic | 206 | 496 | 274 | 218 | 208 | 129 |
| 17 | irAE | Melanoma | | | | nivolumab (2) | myositis | 43 | 753 | 573 | 147 | 61 | 315 |
| 18 | irAE | NA | | | | pembrolizumab + nivolumab (5) | myocarditis | NA | 379 | 338 | 34 | NA | NA |
| 19 | irAE | Melanoma | | | | spartalizumab (1) | hepatitis | 70 | 1042 | 570 | 447 | 65 | 455 |
| 20 | irAE | PML | | | | nivolumab (1) | digestive | 18 | 424 | 304 | 121 | 0 | 17 |
| 21 | irAE | Kidney | | | | nivolumab (1) | myositis/myocarditis | 19 | 535 | 216 | 302 | 87 | 356 |
| 22 | irAE | Kidney | | | | nivolumab + ipilimumab (4) | myeloradiculitis | 22 | 2605 | 1944 | 591 | 602 | 129 |
| 23 | irAE | Lymphoma | | | | pembrolizumab (3) | polyradiculoneuritis | 68 | 497 | 306 | 184 | 344 | 17 |
| 24 | irAE | Melanoma | | | | nivolumab (4) | myocarditis | 84 | 433 | 367 | 68 | 75 | 48 |
| 25 | irAE | Melanoma | | | | nivolumab (1) | myocarditis | 69 | 1755 | 1195 | 462 | 128 | 296 |
| 26 | irAE | Melanoma | | | | nivolumab (1) | myositis | 29 | 516 | 392 | 120 | 31 | 90 |
| 27 | irAE | Kidney | | | | nivolumab (4) | neurologic | 14 | 1376 | 910 | 458 | 451 | 469 |
| 28 | irAE | Melanoma | | | | nivolumab (4) | myositis | 17 | 1172 | 802 | 377 | 343 | 275 |
| 29 | irAE | Bladder | | | | pembrolizumab (3) | pneumopathy | 44 | 306 | 117 | 182 | 78 | 35 |
| 30 | irAE | NA | | | | pembrolizumab (3) | GBS | 15 | 887 | 475 | 447 | 162 | 67 |
| 31 | irAE | Colorectal | | | | pembrolizumab (NA) | encephalitis | NA | 685 | 618 | 62 | 211 | 56 |
| **Median (SD)** | |  |  | | | 3 (3.2) |  | 37 (61) | 535 (589) | 367 (443) | 184 (169) | 125 (165) | 129 (158) |
| **P-value** |  | - | | | | 0.957 | - | 0.016 | 0.596 | 0.503 | 0.441 | 0.974 | 0.528 |

GBS: Guillain Barré Syndrome), PML Progressive multifocal leukoencephalopathy; NA: not available; Patients 8, 13, 17, 23, and 27 were under corticotherapy at the time of blood collection. Mann Whitney test.
